# Spatial distribution and temporal trends of tuberculosis case notifications, Uganda: a ten-year retrospective analysis (2013–2022)

**DOI:** 10.1186/s12879-023-08951-0

**Published:** 2024-01-04

**Authors:** Freda Loy Aceng, Steven Ndugwa Kabwama, Alex Riolexus Ario, Alfred Etwom, Stavia Turyahabwe, Frank Rwabinumi Mugabe

**Affiliations:** 1 Uganda Public Health Fellowship Program, P.O. Box 7072, Kampala, Uganda; 2 National Tuberculosis and Leprosy Program, Ministry of Health, Kampala, Uganda

**Keywords:** Tuberculosis, Case Notification Rate, Spatial distribution, Temporal trends, Uganda

## Abstract

**Background:**

Uganda has a high incidence and prevalence of tuberculosis (TB). Analysis of spatial and temporal distribution of TB is an important tool for supporting spatial decision-making, planning, and policy formulations; however, this information is not readily available in Uganda. We determined the spatial distribution and temporal trends of tuberculosis notifications in Uganda, 2013–2022.

**Methods:**

We conducted a retrospective analysis of routinely-generated program data reported through the National TB and Leprosy Programme (NTLP) surveillance system. We abstracted data on all TB cases diagnosed from 2013 to 2022 by district and region. We drew choropleth maps for Uganda showing the TB case notification rates (CNR) per 100,000 and calculated the CNR using the cases per district as the numerator and individual district populations as the denominators. Population estimates were obtained from the 2014 National Population and Housing Census, and a national growth rate of 3% was used to estimate the annual population increase.

**Results:**

Over the entire study period, 568,957 cases of TB were reported in Uganda. There was a 6% annual increase in TB CNR reported from 2013 (134/100,000) to 2022 (213/100,000) (p-value for trend p < 0.00001). Cases were reported from all 12 Ministry of Health regions during the entire period. The distribution of CNR was heterogeneous throughout the country and over time. Moroto, Napak and Kampala districts had consistently high CNR throughout the ten years. Kalangala district had lower CNR from 2013 to 2018 but high CNR from 2019 to 2022. Moroto region, in the northeast, had consistently high CNR while Mbale and Soroti regions in Eastern Uganda had the lowest CNR throughout the ten years.

**Conclusion:**

There was an overall increasing trend in TB CNR from 2013 to 2022. We recommend that the National TB program institutes intensified measures aided by more funding to mitigate and reverse the negative impacts of the COVID-19 pandemic on TB.

**Supplementary Information:**

The online version contains supplementary material available at 10.1186/s12879-023-08951-0.

## Background

Tuberculosis (TB) is one of the major causes of morbidity and mortality globally, with 10.6 million people estimated to have had TB disease in 2021 [1]. Approximately 1.4 million HIV-negative persons and 187,000 HIV-positive persons died from TB in 2021 [2]. Uganda is one of the 30 countries with the highest burden of TB/HIV, with an estimated TB incidence of 200 cases per 100,000 [3]. In 2018, the proportions of new and previously-treated TB cases caused by multidrug-resistant TB (MDR-TB) and rifampin-resistant TB were estimated at 1% and 12%, respectively [3].

While TB data are often reported as single nation-wide incidence or prevalence, the patterns of TB distribution may be more heterogeneous on local levels [4–7]. The analysis of spatial and temporal distribution of TB can act as a tool for aiding regional planning and influence government policies as well as policy formulations [8].

Data from the 2015 National TB survey in Uganda showed an incidence of TB of 201/100,000 population and prevalence of 253/100,000 population [9]. Although reports show that TB occurs in hot spots in both urban and rural areas [9], there are limited data on district-specific patterns. Identifying districts with high TB prevalence in Uganda can enable improved focus on preventive and control interventions. We determined the spatial distribution and temporal trends of tuberculosis notifications in Uganda, 2013–2022, to provide data to the National TB and Leprosy Programme (NTLP) to develop spatially-targeted interventions.

## Methods

### **Study setting**

In 2022, Uganda had an estimated population of 44,212,800 [10]. The country is divided into four administrative regions (Central, Western, Northern and Eastern), and 12 health regions, which were at the time further subdivided into 112 districts (districts have since increased in number) [11]. As of 2017, Uganda had 155 hospitals, of which 2 were National Referral Hospitals (Mulago and Butabika hospital), 14 were Regional Referral Hospitals (RRHs), and 139 were General Hospitals (GHs) [12]. There are also health centers (HC) II, III, and IV offering different levels of service: HCIII, HCIV, hospitals, and some private HCII facilities have capacity for diagnosis and treatment of TB. The National TB Reference Laboratory (NTRL) oversees the laboratory network throughout the country. Besides NTRL, the network has approximately 1,500 TB diagnostic units, 112 district and 12 regional laboratories, all with capacity to diagnose TB. As of 2016, the network had 1,400 light microscopes, 100 fluorescent microscopes, and 111 XpertMTB/Rif machines for TB diagnosis. At NTRL, both solid and liquid media were used for carrying out drug susceptibility testing (DST) for both first-line and second-line drugs. NTRL is also responsible for transport sputum referral system for routine surveillance of DR-TB and external quality assurance for smear microscopy and for XpertMTB/Rif. In addition, NTRL acts as a supranational TB reference laboratory, the second in Africa, and is currently linked to and supports 11 countries in the region [9].

### Study design and data source

We conducted a retrospective analysis of routinely-generated program data reported through the NTLP surveillance system. People with presumptive TB (defined as a person who presents with signs or symptoms suggestive of TB) are recorded in the presumptive TB registers. Clinicians send these people with TB to the laboratory based on clinical examination. People with bacteriologically confirmed TB are recorded in the laboratory register, which is later copied into the unit TB register. Health facility in-charges report the TB cases to the district. From the health facility, the District TB and Leprosy Supervisors record in the District TB register, from which quarterly reports are generated using a standard reporting template developed by the NTLP. The NTLP uses those data for planning.

We included all TB cases registered by the health services from 2013 to 2022 across all health regions.

### Study variables, data management, and analysis

We entered data in MS Excel spreadsheets and exported to Epi Info version 7.2.0 for analysis. We calculated the TB CNR per 100,000 per year by district and region. Population estimates were obtained from the 2014 National Population and Housing Census, and a national growth rate of 3% was used to estimate the yearly population increase [11].

We used logistic regression [13] to determine the percentage annual change in CNR over the study period, as follows:

$$ Logit \left(P\right)=log\left(\frac{p}{1-p}\right)=\alpha +\beta x+\epsilon $$,

$$ p\left(x\right)=\frac{1}{1+{e}^{-(\alpha +\beta x)}}OR=\frac{{e}^{\alpha +\beta (x+1)}}{{e}^{\alpha +\beta \left(x\right)}}={e}^{\beta }$$where $$ p\left(x\right)$$ is CNR for a given year ($$ x$$), $$ \alpha $$ is the logistic regression intercept, $$ \beta $$ is the logistic regression coefficient for year ($$ x$$), and $$ \epsilon $$ is the error term. The statistical significance of $$ \beta $$ was used to assess the significance of the temporal trends of CNR, and the odds ratio ($$ OR$$) was used to estimate the average annual percentage change in CNR.

To describe the spatial distribution of TB CNR, we used QGIS (Quantum Geographic Information System) software. We drew choropleth maps for Uganda showing the TB CNR per 100,000.

## Results

**Fig. 1:**
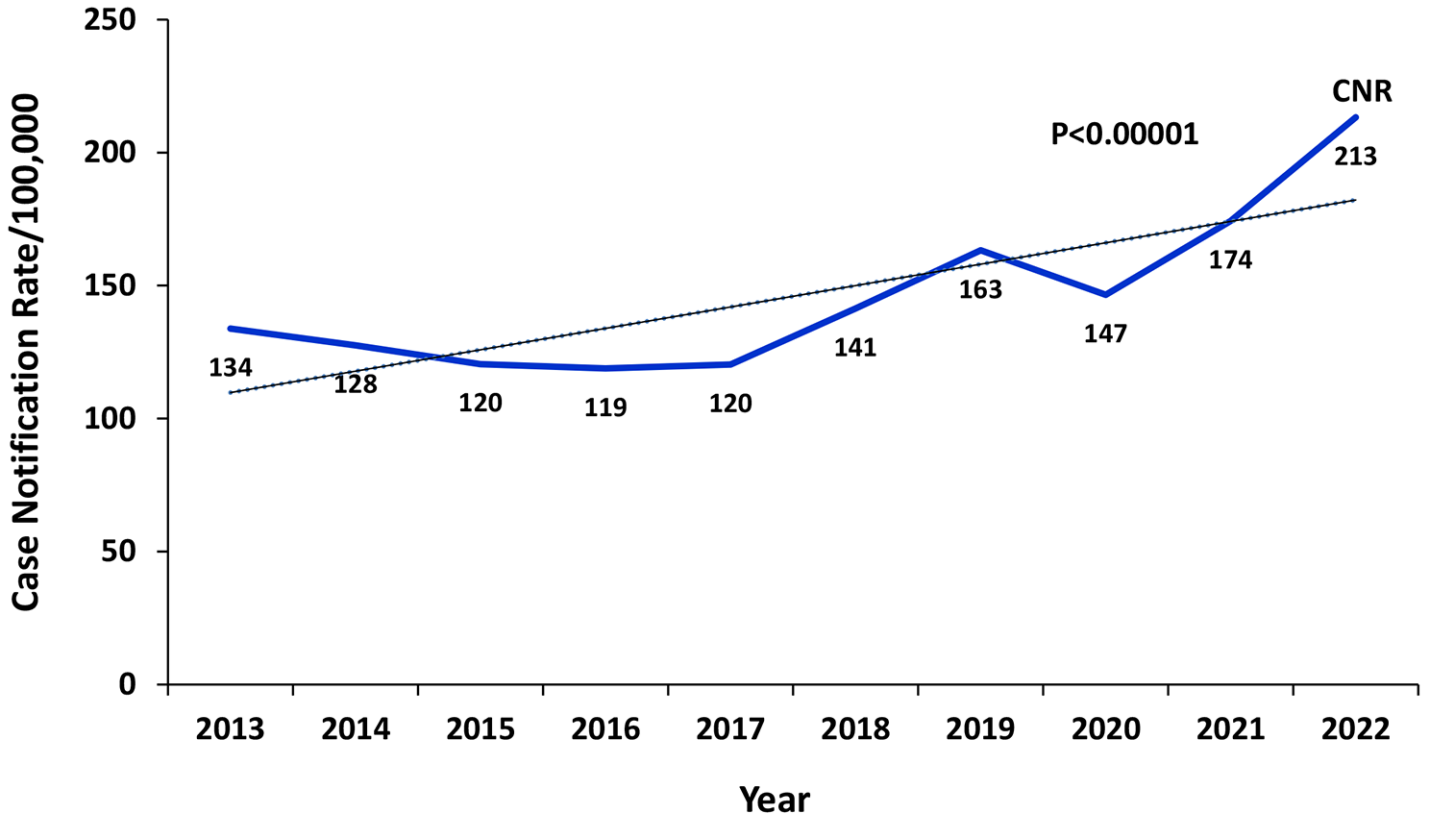
Tuberculosis Case notification rates per 100,000, Uganda, 2013–2022

Over the entire study period, 568,957 cases of TB were reported. There were 44,886 cases in 2013 and 94,286 cases in 2022. There was a 6% annual increase in TB CNR reported from 2013 (134/100,000 population) to 2022 (213/100,000 population) (p-value for trend p < 0.00001). Overall, case notification rates increased during this period (Fig. 1). Between 2013 and 2017 there was a decline in TB CNR from 134/100,000 to 120/100,000. There was an increase in TB CNR from 120/100,000 in 2017 to 163/100,000 in 2019. From 2019 there was a decrease in TB CNR to 147/100,000 in 2020. From 2020 there was an increase in TB CNR to 213/100,000 in 2022.

Cases were reported from all 12 Ministry of Health regions during the entire period.

**Fig. 2:**
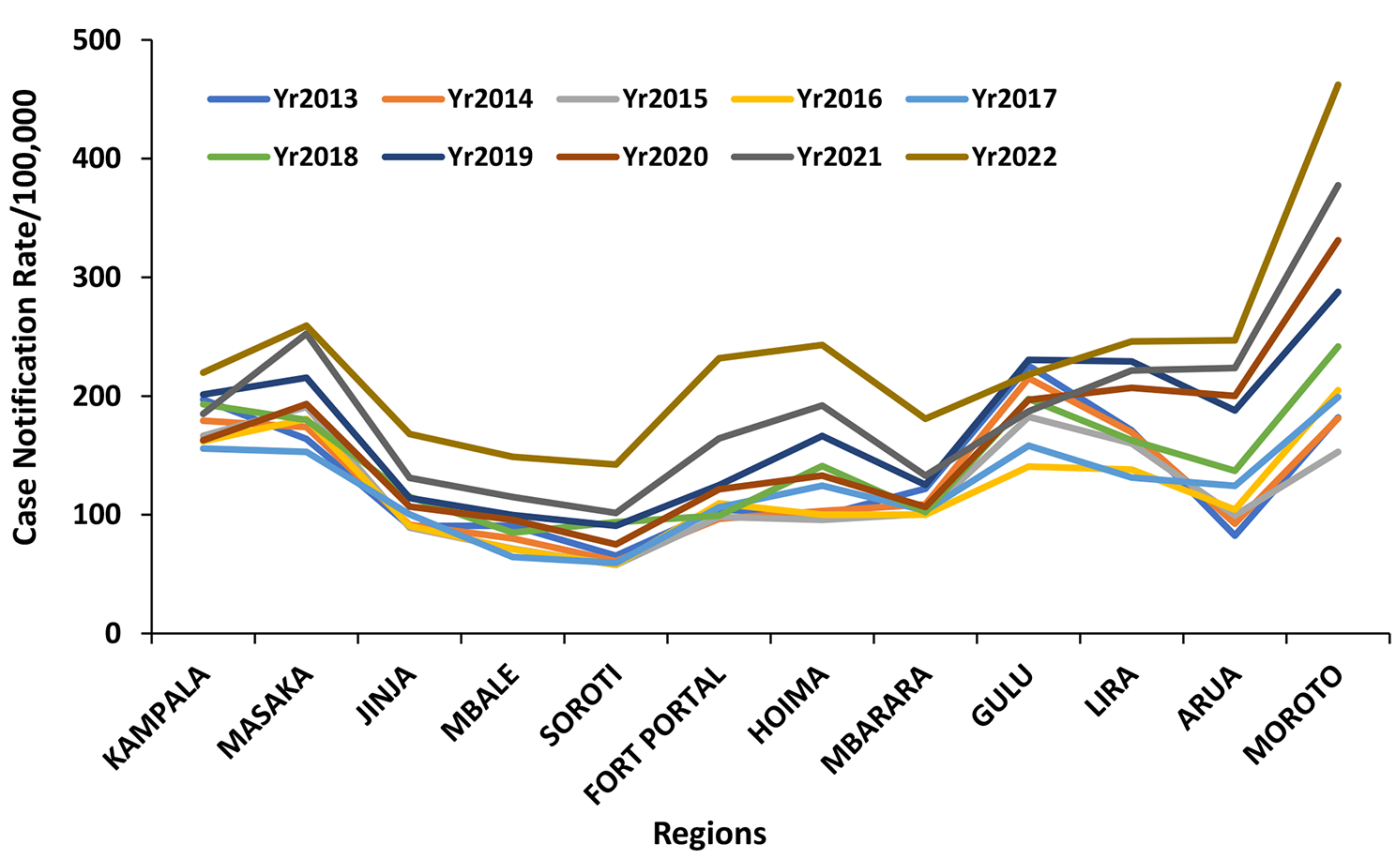
Regional Tuberculosis Case notification rates per 100,000, Uganda, 2013–2022

**Fig. 3:**
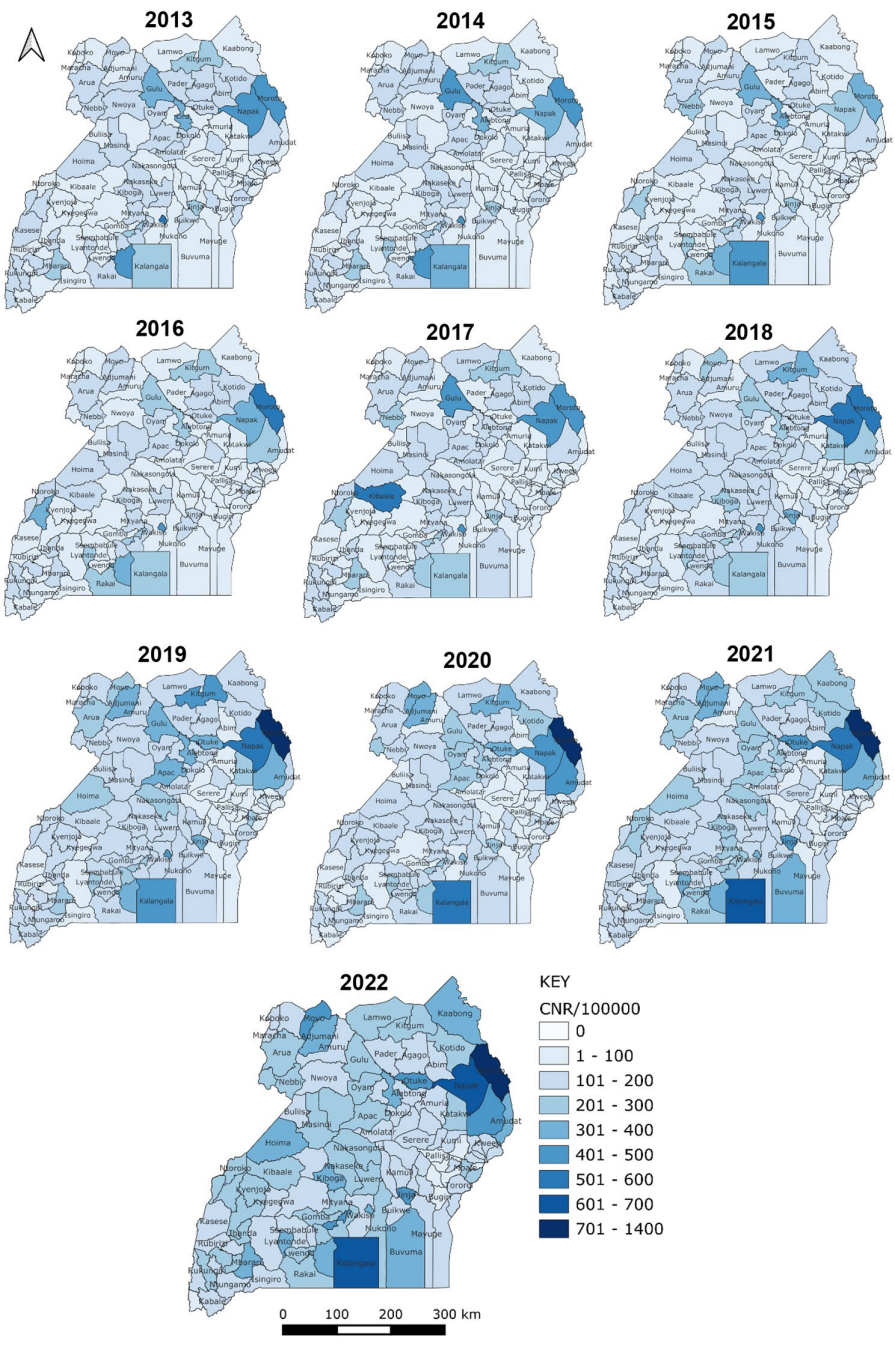
Tuberculosis Case Notification Rates per 100,000, Uganda Districts, 2013–2022

The regions showed variable CNR across the ten-year period. Moroto region, in the northeast, had consistently high CNR throughout the ten years while Mbale and Soroti regions in Eastern Uganda had the lowest CNR throughout the ten years (Fig. 2). Kampala and Masaka districts had high but declining CNR throughout the study period, while Moroto, Napak and Kampala districts had consistently high, though varying, CNRs. Kalangala district had increasing CNR over the period, from 283 in 2013 to 663 in 2022. Bududa, Bugiri, Kibuku, Namutumba and Pallisa districts had low CNR throughout the ten years (Fig. 3).

## Discussion

We identified an increase in overall reported cases of tuberculosis in Uganda during 2013–2022. The distribution of CNR was heterogeneous throughout the country. This is similar to a study in Ethiopia that found variations in CNR [14]. There was a fluctuation in CNRs throughout the country for the ten years. This can be attributed to several factors such as the health-seeking behavior of people with TB, resource availability, health worker motivation as well as incomplete and inaccurate reporting.

In this study, the overall increase is likely due to a combination of factors including a longstanding national TB control program, the presence of at least one focal person in every district and technical and financial support from implementing partners distributed in various districts. In addition, the increase might be attributed to improved reporting and capacity to diagnose TB.

Over five years (2013–2017), there was a decline in CNR. Declines in case notifications are due either to real declines in TB cases in the population, changes in the diagnosis rates, or changes in the notification rates. The decrease could be due to under-reporting and limited capacity to diagnose TB. This could have been due to low utilization of GeneXpert testing because of knowledge gap on eligibility criteria and low index of suspicion [15]. Our findings are similar to a study in Kenya which identified declining temporal trends over five years [16]. The program expectation is for an increase in the CNR because the current priority is to find missed TB cases. However, there were barriers hence the decrease in CNRs.

TB case notification rates declined over that period. The country’s performance in TB case detection and treatment outcome indicators had stagnated over the years. According to the 2016/17 annual report, approximately 40% of the estimated incident TB cases were missed and there was successful treatment of only 80% of the previous year’s incident cases [17].

There was an increase in CNR between 2016 and 2017 from 119/100,000 population to 120/100,000 population. Following the 2015 National TB Prevalence survey, the NTLP devised new strategies to obtain the missing TB cases such as strengthening TB screening among contacts, use of radiological investigations among asymptomatic people with TB, and acquisition of more GeneXpert machines. These interventions are what probably led to the increase in notified TB cases in that time period.

Active case finding, which refers to screening whole communities for active TB, would be the ideal way of finding nearly all the TB cases including those currently being missed. However, the major challenge is that it would be expensive. The alternative is to screen high TB risk groups which would lead to finding a good number of TB cases but not as many as would be found through active case finding. However, depending on where and how screening high TB risk groups is done it would also still be quite expensive [17, 18]. In Uganda, ACF began in 2018 and it is not surprising that the TB CNR has been consistently higher than the pre-ACF period.

The COVID-19 pandemic continues to have a negative impact on access to TB diagnosis and treatment and the burden of TB disease. Globally, there was a large drop in the reported number of people newly diagnosed with TB in 2020 [2]. In our study, the decline in TB CNR in 2020 can be attributed to the COVID-19 pandemic which disrupted healthcare worldwide [19, 20]. During this time, measures that were executed to curb the spread of COVID-19 such as banning of public transport and eventually lock down may have interfered with the population health seeking behavior hence a decline in TB diagnoses [21]. Our findings are similar to a study in Eswatini that found a significant decrease in TB CNR during the COVID-19 pandemic [22]. The increase in TB CNR in 2021 and subsequently 2022 suggests that the number of people with undiagnosed and untreated TB grew during the intense waves of the COVID-19 pandemic resulting in more community transmission of infection and hence increased numbers of people developing TB [2].

Also, later during the COVID-19 response there was a pillar known as the Continuity of Essential Health Services which ensured that TB diagnosis and treatment continued on a more stable basis through integration of TB and COVID-19 screening. In 2021, a community TB catch-up campaign was done in high burden districts to increase case detection and create community awareness about TB and this led to an increase in TB case notifications [23].

In 2022, the National TB and Leprosy Program with support from partners began the Community Awareness, Screening, Testing, Prevention and Treatment Approach to Ending TB and Leprosy (CAST TB/Leprosy) campaign. This is a biannual countrywide approach that focuses on igniting communities for the uptake of preventive and treatment innovations for TB services through community participation. It was designed to help the country catchup with the missed TB and Leprosy diagnoses during the COVID-19 pandemic and it has resulted into an increase in TB CNR [23, 24].

Cases were reported from all 12 Ministry of Health regions during the entire time period. The regions showed variable CNR throughout the ten-year period. Moroto region showed consistently higher CNR throughout the ten years. This could be due to the presence of implementing partners such as Doctors with Africa that support better diagnosis. Previous studies have shown TB spatial variability across study regions [8]. The Moroto region is comprised of a nomadic population and characterized by poor housing where they live in clusters, chronic malnutrition, drought and semi-illiteracy which facilitate TB progression to disease hence more people affected with TB. Of the seven districts in the Moroto region, Moroto and Napak districts had consistently high TB CNR and this can be attributed to the presence of the two major hospitals in the region, Moroto Regional Referral Hospital and Matany hospital respectively that handle a large volume of people with TB. Kampala district which is the capital city of Uganda also had consistently high TB CNR throughout the ten years. This could be because it has the National Referral Hospital therefore many people with TB from other parts of the country seek healthcare from there. This is similar to findings from a study in Ethiopia that found high urban TB CNR [25]. Mbale and Soroti regions consistently had the lowest CNR throughout the ten years and this could be because the regions were not implementing programmatic strategies efficiently, poor screening, low reporting or actual low prevalence of TB in the region.

Uganda has made tremendous efforts to lower the HIV burden through interventions that improve antiretroviral therapy (ART) uptake hence this translates into better health among the HIV infected hence lowering the TB prevalence. This scenario is similar to a study in Kenya that attributed the decline of TB in part to significant efforts made by the country to address the burden of HIV among people with TB with increased cotrimoxazole preventive therapy (CPT) and ART uptake [26]. The 2016 Uganda Population HIV Impact Assessment (UPHIA) showed a fall in HIV national prevalence at 6% compared to 7.3% according to the 2011 Uganda AIDS Indicator Survey. The decline in HIV prevalence is due to the intensified HIV prevention and treatment services in the country that lead to a decrease in the number of new infections in recent years [27].

The major limitation with our study was the use of secondary data which had some inconsistencies in recording and reporting likely leading to an underestimation of the CNR. Also, the data used was aggregated hence it was not possible to analyze additional variables such as underlying clinical conditions. In addition, using retrospective data makes it difficult to more specifically explain the observed trends. However, in light of national programs, the trends have been explained.

## Conclusion

There was an overall increasing trend in TB CNR from 2013 to 2022. A slight increase in TB CNR that occurred in 2017 could have resulted in increase in TB case notification to the NTLP following results of the 2015–2016 prevalence survey that showed almost 50% of TB cases missed annually. We therefore recommend that the National Tuberculosis and Leprosy program should institute intensified measures aided by more funding to identify cases of TB, mitigate and reverse the negative impacts of the COVID-19 pandemic on TB.

### Electronic supplementary material

Below is the link to the electronic supplementary material.

Supplementary Material 1

Supplementary Material 2

## Data Availability

The datasets used and analyzed during this study belong to the Uganda Public Health Program and are not publicly available. However, the datasets could be availed by the corresponding author upon reasonable request and with permission from the Uganda Public Health Fellowship Program.

## Abbreviations

ART: Anti-Retroviral Therapy
CDC: Centers for Disease Control and Prevention
CNR: Case Notification Rate
GHs: General Hospitals
MDT: Multidrug therapy
NTLP: National Tuberculosis and Leprosy Program
PFP: Private for-profit
PNFP: Private not-for-profit
QGIS: Quantum Geographic Information System
RRHs: Regional Referral Hospitals
WHO: World Health Organisation
