# Supplementary material for: Spatial distribution and temporal trends of tuberculosis case notifications, Uganda: a ten-year retrospective analysis (2013–2022)
Source: BMC Infect Dis. 2024 Jan 4;24:46. doi: 10.1186/s12879-023-08951-0 (PMC10765632; doi:10.1186/s12879-023-08951-0)
Supplement: Supplementary file 1 — Supplementary Material 1 [file 12879_2023_8951_MOESM1_ESM.docx]

**Table 1: Regional Tuberculosis Case notification rates per 100,000, Uganda, 2013-2022**

| **YEAR** | **2013** | **2014** | **2015** | **2016** | **2017** | **2018** | **2019** | **2020** | **2021** | **2022** |
| --- | --- | --- | --- | --- | --- | --- | --- | --- | --- | --- |
| **KAMPALA** | 197 | 179 | 167 | 162 | 156 | 193 | 201 | 163 | 185 | 220 |
| **MASAKA** | 164 | 174 | 191 | 181 | 153 | 180 | 216 | 193 | 253 | 259 |
| **JINJA** | 91 | 92 | 89 | 91 | 100 | 115 | 114 | 107 | 131 | 168 |
| **MBALE** | 91 | 80 | 71 | 71 | 64 | 85 | 100 | 96 | 115 | 149 |
| **SOROTI** | 65 | 61 | 58 | 58 | 59 | 94 | 91 | 75 | 102 | 142 |
| **FORT PORTAL** | 105 | 97 | 98 | 110 | 106 | 99 | 125 | 122 | 164 | 232 |
| **HOIMA** | 99 | 103 | 96 | 100 | 124 | 141 | 166 | 133 | 192 | 243 |
| **MBARARA** | 122 | 109 | 101 | 100 | 103 | 102 | 125 | 107 | 133 | 181 |
| **GULU** | 226 | 215 | 182 | 141 | 158 | 198 | 231 | 197 | 187 | 218 |
| **LIRA** | 171 | 170 | 160 | 138 | 131 | 163 | 229 | 207 | 222 | 246 |
| **ARUA** | 82 | 93 | 99 | 104 | 124 | 137 | 188 | 200 | 224 | 247 |
| **MOROTO** | 182 | 181 | 153 | 205 | 199 | 242 | 288 | 331 | 378 | 462 |
